# Scalable (Enantioselective) Syntheses of Novel 3-Methylated Analogs of Pazinaclone, (*S*)-PD172938 and Related Biologically Relevant Isoindolinones

**DOI:** 10.3390/molecules27175647

**Published:** 2022-09-01

**Authors:** Antonia Di Mola, Giorgia Nicastro, Lorenzo Serusi, Rosanna Filosa, Mario Waser, Antonio Massa

**Affiliations:** 1 Dipartimento di Chimica e Biologia “A. Zambelli”, Università degli Studi di Salerno, Via Giovanni Paolo II, 84084 Fisciano, Italy; 2 Dipartimento di Scienze e Tecnologia, Università degli Studi del Sannio, Via De Sanctis, 82100 Benevento, Italy; 3 Institute of Organic Chemistry, Johannes Kepler University Linz, Altenbergerstr. 69, 4040 Linz, Austria

**Keywords:** heterocycles, asymmetric synthesis, isoindolinones

## Abstract

Herein, we report the application of an efficient and practical K_2_CO_3_ promoted cascade reaction of 2-acetylbenzonitrile in the synthesis of novel 3-methylated analogs of Pazinaclone and PD172938, belonging to isoindolinones heterocyclic class bearing a tetrasubstituted stereocenter. Organocatalytic asymmetric synthesis of the key intermediate and its transformation into highly enantioenriched 3-methylated analog of (*S*)-PD172938 was also developed. These achievements can be of particular interest also for medicinal chemistry, since the methyl group is a very useful structural modification in the rational design of new and more effective bioactive compounds.

## 1. Introduction

**Figure 1:**
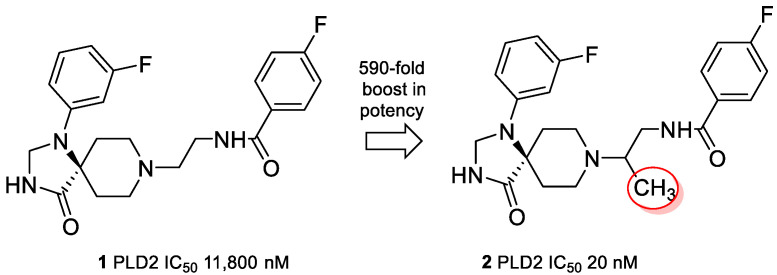
Example of CH methylation where a magic methyl effect was observed.

The importance of methyl groups in biologically active molecules has been recently highlighted, showing that more than 80% of small-molecule drugs contain at least one methyl group bound to a carbon atom [1,2,3,4,5,6,7,8,9,10,11,12,13]. The introduction of a methyl group is used to optimize many properties of a drug candidate, and it is often associate in drug discovery to the so-called magic methyl effect. This strategy can have a favorable effect on solubility or selectivity against off-targets or can allow one to convert an agonist into an antagonist or a partial antagonist into a negative allosteric modulator [5]. It can induce a conformational change up to 590-fold boosts in potency [1]. For example, in the PLD2 inhibitor **2**, the new methyl group improves potency from 11,800 to 20 nM (Figure 1) but adds very little lipophilicity (ΔclogP = 0.36) [1,12]. The introduction of the methyl group has the advantage that only a small change of the physical properties of a molecule (ΔMW = 14 gmol^−1^, ΔclogP = 0.5) is obtained when compared to the trifluoromethyl group (ΔMW = 68 g mol^−1^ and ΔclogP = 0.9), avoiding the formation of too lipophilic molecules and consequently the violation of Lipinsky’s rules [13].

In contrast to the CF_3_ (trifluoromethyl group), which has received much attention from the synthetic community in the past decade, the incorporation of a methyl group represents a significant challenge in many contexts, especially when it must be installed on an existing tertiary stereocenter. Therefore, synthetic protocols that can incorporate the methyl group at specific points in a structure–activity relationship (SAR) program are of high value to medicinal chemistry and to the pharmaceutical industry [2].

**Figure 2:**
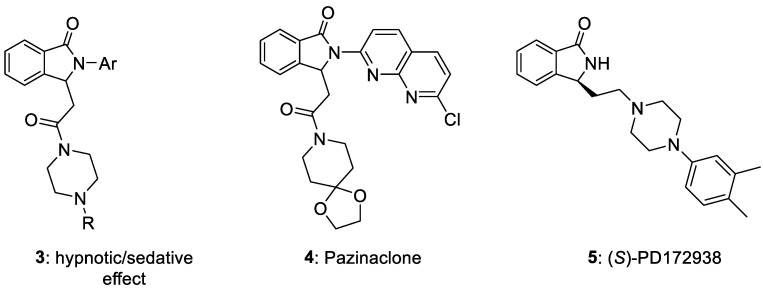
Some bioactive isoindolinones.

Over the past few years, the isoindolinone ring system has emerged as a valuable pharmacophore, exhibiting a wide range of therapeutic activities including antimicrobial, antioxidant, antifungal, anti-Parkinson, anti-inflammatory, antipsychotic, antihypertensive, anesthetic, vasodilatory, anxiolytic and antiviral activities [14,15]. In particular, isoindolinones **3** [16,17,18] and **4** (Pazinaclone) [19,20] are benzodiazepine-receptor agonists for the treatment of anxiety, while compound (*S*)-**5**, developed by Belliotti et al., known as (*S*)-PD172938, is a potent dopamine D_4_ ligand (Figure 2) [21,22].

The incorporation of a methyl group installed directly on the stereocenter of the isoindolinone ring can be of particularly importance for the effect on the binding activity and affinity to the receptors. However, the construction of the isoindolinone ring with a tetrasubstituted carbon stereocenter is a major challenge, especially when this has to be asymmetrically performed [14,23,24]. To our knowledge there are no examples in literature of 3-methylated analogs of the compounds described in Figure 2 and very few protocols on the synthesis of 3,3-disubstituted isoindolinones introducing a methyl group on the C-3 position have been reported [14,25,26,27,28,29,30,31,32,33,34]. For instance, as described by Heibel in 2005, an asymmetric synthesis of 3,3-disubstituted isoindolinones involved the use of readily available (+) or (−)-trans-2-(α-cumyl) cyclohexanol (TCC) as the chiral auxiliary after the treatment with NaHMDS at temperatures ranging between −100 °C and −115 °C leading to good diastereoselectivity [25]. Later Cramer disclosed a chiral CpRh(III)-catalyzed C-H functionalization to directly provide isoindolinones bearing a tetrasubstituted stereocenters with high enantioselectivity [26]. In 2017, Liu utilized a Rh (III)-catalyzed [4 + 1] cyclization reaction of benzamides with propargyl alcohols to prepare isoindolinones bearing a quaternary carbon (with ketones in lateral chains) under external oxidant-free conditions [27]. Recently, a nickel-catalyzed reductive dicarbofunctionalization of 1,1-disubstituted enamides with inactivated alkyl iodides was found effective to access *N*-benzyl-3,3-dialkyl-substituted isoindolinones frameworks in the racemic and asymmetric version [28]. In addition, Watson developed tandem aza-Heck-Suzuki and aza-Heck-carbonylation reactions of O-phenyl hydroxamic ethers to a variety of complex chiral γ-lactams, using highly toxic carbon monoxide gas [29]. However, all these methodologies have several drawbacks, such as harsh reaction conditions, low scale set up and, most importantly, they are not suitable for the synthesis of 3-methylated analogs of the bioactive compounds of Figure 2. In this context, we report a convenient approach to 3,3-disubstituted isoindolinones via cascade reactions of readily available 2-acylbenzonitriles with several nucleophiles promoted by cheap and environmentally friendly K_2_CO_3_ [30,31,32]. Asymmetric versions of these reactions have also been developed [33].

**Scheme 1:**
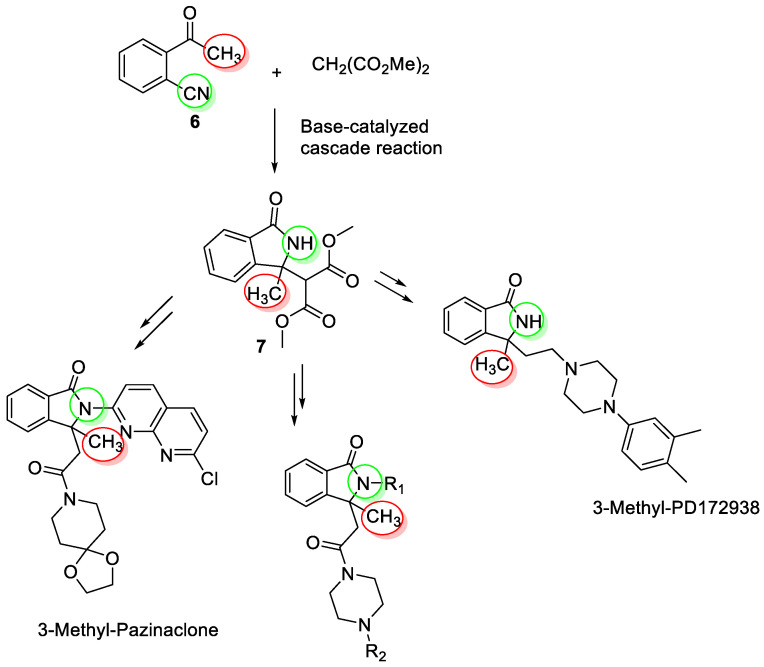
This work: new isoindolinones bearing a methyl group installed in 3-position.

Based on our continuing research interest in the synthesis of isoindolinones [31,32,33,34,35], we report the application of our efficient and practical K_2_CO_3_ promoted a cascade reaction to the first synthesis of 3-methylated analogs of Pazinaclone, PD172938 and related structures (Scheme 1). An efficient asymmetric synthesis of 3-methylated analog of (*S*)-PD172938 was also developed based on the combination of the asymmetric-phase transfer catalysis process and a recrystallization step.

## 2. Results

### 2.1. Synthesis of Novel Racemic 3-Methylated Analogs of Bioactive Isoindolinones

**Scheme 2:**
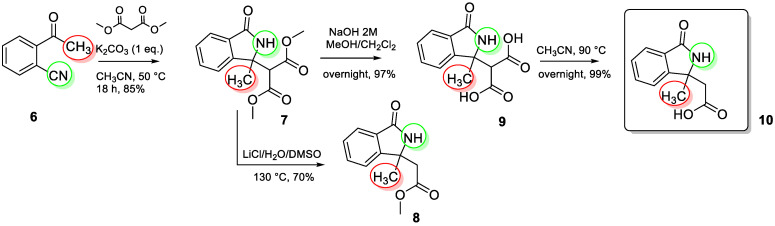
Synthesis of key intermediate **10**.

The first part of the investigation focused on the large-scale synthesis of 3-substituted isoindolinone **7**, which was conveniently obtained in the presence of the K_2_CO_3_-promoted cascade reaction of readily available 2-acetylbenzonitrile **6** and dimethylmalonate in acetonitrile (Scheme 2). This compound proved to be particularly convenient in the synthesis of a series of novel analogs of bioactive 3-substituted isoindolinones, based on the transformation of **7** into the monocarboxylic derivative **10**. For this purpose, several methods were investigated. Decarboxylation of **7** under reflux in 6M HCl solution led to complex mixtures of products, while the treatment of **7** with a LiCl/H_2_O/DMSO mixture at 130 °C, a method known as Krapcho decarboxylation [36,37], allowed for obtaining the ester **8** in a 70% yield. However, a higher yield of **10** was achieved in a two-step process comprising a saponification followed by simple heating of the dicarboxylic acid **9** in CH_3_CN (Scheme 2).

**Scheme 3:**
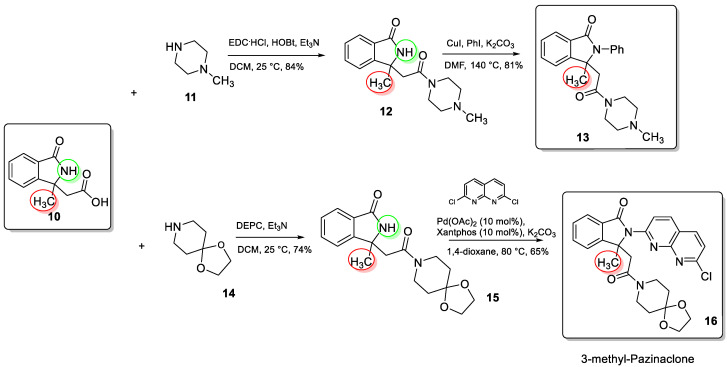
Synthesis of racemic amidic compounds **13** and **16**.

The key intermediate **10** was, then, easily converted in the target isoindolinones according to the strategies described in Scheme 3. Amide **12** derived from *N*-methylpiperazine **11** was easily obtained by EDC/HOBt activation of the carboxylic group. In the case of 1-piperidone ketal **14**, Yamada coupling using DEPC (diethylphosphoryl cyanide) was more efficient [38].

NH arylation of the lactams **12** and **15** required several efforts too (Scheme 3). In contrast with arylation of 3-mono-substituted isoindolinones [22,30], phenylation of the lactam **12** was performed using a ligand-less cross coupling reaction based on the Cu(I)-catalyzed arylation of amides [39] in a very good 81% yield, allowing for access to the 3-methylated analog **13** of a hypnotic sedative drug (Figure 2). In the synthesis of 3-methyl-Pazinaclone **16**, the palladium-catalyzed Buchwald–Hartwig reaction of the lactam **15** in the presence of Xantphos [40] was necessary with the less reactive 1,8-dichloronaphthiridine, while the use of BINAP as ligand or Cu(I)-catalyzed arylation reactions were not effective.

**Scheme 4:**
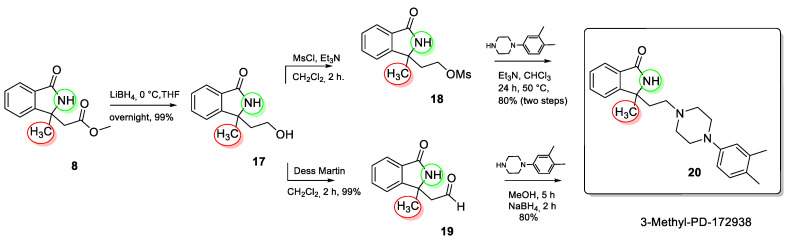
Synthesis of 3-methyl-PD172938 in racemic form.

The target compound **20**, 3-methyl-PD172938, was obtained by two different pathways as described in Scheme 4:

(a) Displacement reaction of the mesyl group in **18** by 3,4-dimethylphenylpiperazine;
(b) Reductive amination of the aldehyde **19** with 3,4-dimethylphenylpiperazine.

First of all, ester **9** was chemoselectively reduced by LiBH_4_ in quantitative yield. Subsequent mesylation of the alcohol **17** and displacement of the mesyl group with the 3,4-dimethylphenylpiperazine led to 3-methyl-PD172938 **20** in a high overall yield. For this purpose, it has to be noted that the sequential one-pot mesylation/piperazine displacement gave sluggish results (addition of piperazine at the end of mesylation reaction). Purification of **18** on silica gel led to decomposition products, while purification on Florisil afforded **18** in a good 73% yield. However, the best results were obtained treating the crude product **18** with 3,4-dimethylphenylpiperazine at 50 °C in CHCl_3_, leading to the target 3-methyl-PD172938 **20** with 80% overall yield for two consecutive steps. Alternatively, compound **20** can be afforded with similar level of efficiency by reductive amination of the aldehyde **19** with NaBH_4_ in the presence of 3,4-dimethylphenylpiperazine. The aldehyde **19** is readily accessible in quantitative yield by Dess–Martin oxidation of the alcohol **17**, while the reduction of **8** in **19** was not attempted since DIBAL-H is not selective in the presence of amides [41].

### 2.2. Asymmetric Synthesis of 3-Methylated Analog of (S)-PD172938

**Scheme 5:**
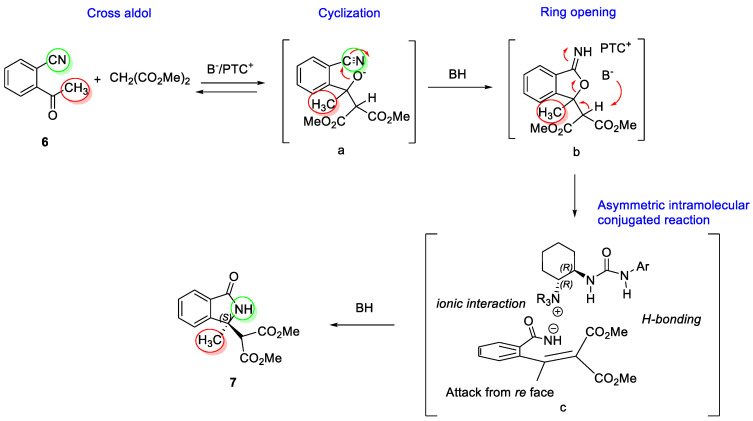
Proposed mechanism for the base-catalyzed cascade reaction of 2-acetylbenzonitrile and dimethylmalonate.

**Scheme 6:**
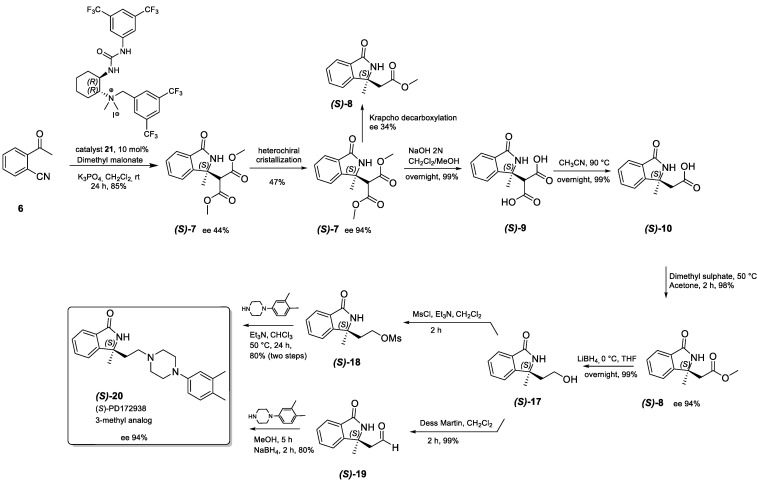
Enantioselective synthesis of (*S*)-**20**, the 3-methylated analog of (*S*)-PD172938.

Then, the asymmetric synthesis 3-methylated analog of (*S*)-PD172938 was developed. For direct applications in medicinal chemistry programs, the ability to selectively construct a new stereocenter with enantiocontrol is of great importance. Since convenient methods for the asymmetric synthesis of the key intermediate **8** and related compounds bearing a tetrasubstituted stereocenter are not available, we considered the previously investigated asymmetric cascade reaction of readily available 2-acetylbenzonitrile and dimethylmalonate for the direct access to enantioenriched **7** (Scheme 5 and Scheme 6) [33]. The development of an effective asymmetric version of this cascade reaction proved to be particularly challenging. A large number of chiral neutral bifunctional organocatalysts and chiral ammonium salts were tested under a range of conditions [33]. The best results were obtained in the presence of the chiral bifunctional ammonium salt **21**-derived form (*S,S*)-1,2-cyclohexanediamine and K_2_CO_3_ as the inorganic base in CH_2_Cl_2_ under phase transfer conditions (Scheme 5 and Scheme 6), leading to moderate enantioselectivity [33]. The possibility to easily synthesize catalyst **21** in high scale [42] allowed us to conveniently scale up the cascade process to 1.72 mmol of 2-acetylbenzonitrile. Despite the moderate enantioselectivity also achieved on a large scale, the advantage of the entire process relies on a very effective and reproducible process of enantio-enrichment by crystallization, increasing the ee of **7** up to 94% in an acceptable overall yield (Scheme 6). In addition, the chiral ammonium salt **21** was recovered after the chromatography in 75% yield and reused with similar level of enantioselectivity, with the possibility to improve the efficiency of the process. The absolute configuration of compound (+)-**7** was previously established to be (*R*) by VCD measurements when (*S,S*)-**21** is used [33]. This is an important finding since in the present investigation, we used (*R,R*)-**21** to synthesize **7** with (*S*) configuration. The knowledge of absolute configuration allowed us to propose a plausible transition state for the enantioselective determining step (Scheme 5). A combination of both ionic and hydrogen bond interactions with **21** should favor the intramolecular aza-Michael reaction from the *re* face of prochiral intermediate **c** leading to **7** (Scheme 5). The mechanism of the reaction is proposed based on previous experimental [31,33] and theoretical studies [32]. After deprotonation of dimethylmalonate, the resulting carboanion leads to carbonyl addition to ketone, followed by cyclization at cyano group and formation of the cyclic imidate **b**, which will give then rearrangement to the final product **7**.

The synthesis of (*S*)-**10** was successfully achieved by the two-step decarboxylation of (*S*)-**7** without decrease of the enantiomeric purity (Scheme 6). Krapcho decarboxylation was firstly attempted because it directly leads to the ester **8** in high yield. However, the product was recovered with significant racemization (34% ee) probably due to reversible ring opening/ring closing via the retro-Michael reaction eventually occurring at the high temperature. The target compound (*S*)-**20**, 3-methylated analog of (*S*)-PD172938, was obtained by the two different pathways as previously described for racemate synthesis: via the mesyl group displacement in (*S*)-**18** or reductive amination of aldehyde (*S*)-**19**, without loss of enantiopurity in both the cases (94% ee) and acceptable overall yields, as described in Scheme 6.

## 3. Experimental Section

### 3.1. General Information

Unless otherwise noted, all chemicals, reagents and solvents for the performed reactions are commercially available and were used without further purification. 2-Acetylbenzonitrile was purchased from Fluorochem. 1,8-Dichloronaphthiridine was prepared according to the literature [43,44]. Catalyst **21** was synthesized according to the procedure described in [42]. All the reactions were monitored by thin layer chromatography (TLC) on precoated silica gel plates (0.25 mm) and visualized by fluorescence quenching at 254 nm. Flash chromatography was carried out using silica gel 60 (70–230 mesh, Merck, Darmstadt, Germany). Yields are given for isolated products showing one spot on a TLC plate, and no impurities were detectable in the NMR spectrum. The NMR spectra were recorded on Bruker DRX 600, 400 and 300 MHz spectrometers (600 MHz, ^1^H, 150 MHz, ^13^C; 400 MHz, ^1^H, 100.6 MHz, ^13^C; 300 MHz, ^1^H, 75.5 MHz, ^13^C; 250 MHz, ^1^H, 62.5 MHz, ^13^C). Internal reference was set to the residual solvent signals (δ_H_ 7.26 ppm, δ_C_ 77.16 ppm for CDCl_3_). The ^13^C NMR spectra were recorded under broadband proton decoupling. Spectra are reported only for unknown compounds. The following abbreviations are used to indicate the multiplicity in NMR spectra: s—singlet, d—doublet, t—triplet, q—quartet, dd—doublet of doublets, m—multiplet and brs—broad signal. Coupling constants (J) are quoted in Hertz. High-resolution mass spectra (HRMS) were acquired using a Bruker SolariX XR Fourier transform ion cyclotron resonance mass spectrometer (Bruker Daltonik GmbH, Bremen, Germany) equipped with a 7T refrigerated actively shielded superconducting magnet. For ionization of the samples, electrospray ionization (ESI) or MALDI was applied. Enantiomeric excess of chiral compounds was determined by HPLC using chiral column.

### 3.2. Dimethyl 2-(1-Methyl-3-Oxoisoindolin-1-yl) Malonate (***7***)

To a solution of 2-acetylbenzonitrile **6** (500 mg, 3.44 mmol, 1.0 eq.) in dry CH_3_CN (1.2 mL), potassium carbonate (476 mg, 3.44 mmol, 1.0 eq.) and dimethylmalonate (1.5 mL, 10 mmol, 3.0 eq.) were added. The mixture was stirred at 50 °C for 24 h, then diluted with chloroform and filtered. Purification by crystallization (Dichloromethane/Hexane, 5 mL/12.5 mL) a −20 °C for 24 h afforded the pure product, as a yellow solid. Yield: 85% (809 mg). HRMS (ESI) *m/z:* [M + H]^+^ calcd. for C_14_H_16_NO_5_ 278.10230; found: 278.10280. ^1^H NMR (300 MHz, CDCl_3_): δ 7.80 (d, *J* = 7.38 Hz, 1H), 7.58–7.50 (m, 1H), 7.46 (t, *J* = 7.50 Hz, 1H), 7.38 (d, *J* = 7.59 Hz, 1H), 7.20 (brs, 1H), 3.84 (s, 1H), 3.79 (s, 3H), 3.43 (s, 3H), 1.68 (s, 3H). M.p and spectroscopic data were in agreement with the literature [31].

### 3.3. Methyl 2-(1-Methyl-3-Oxoisoindolin-1-yl) Acetate (***8***)

In a Schlenk tube, anhydrous lithium chloride (180 mg, 4.0 eq.) was added to a solution of dimethyl 2-(1-methyl-3-oxoisoindolin-1-yl) malonate **7** (300 mg, 1.10 mmol, 1.0 eq.) in DMSO (5.0 mL) and water (500 µL). The reaction mixture was stirred at 130 °C until the starting material disappeared by thin-layer chromatography (CHCl_3_/AcOEt,1:4). The reaction mixture was diluted with ethyl acetate and washed with brine, and then the organic phase was dried on anhydrous sodium sulphate and concentrated under reduced pressure to give an oil. Purification by flash chromatography on silica gel (Hexane/AcOEt 50/50) gave the pure product as a yellow wax-type oil. Yield: 70% (240 mg). IR (KBr): 3201, 1720, 1680 cm^−1^. HRMS (ESI) *m/z:* [M + H]^+^ calcd for C_12_H_14_NO_3_ 220.09682, found: 220.09679. ^1^H NMR (400 MHz, CDCl_3_): 7.83 (d, *J* = 7.53 Hz, 1H), 7.57 (t, *J* = 7.39 Hz, 1H), 7.47 (t, *J* = 7.49 Hz, 1H), 7.37 (d, *J* = 7.56 Hz, 1H), 7.14 (bs, 1H), 3.72 (s, 3H), 2.96 (d, *J* = 16.3 Hz, 1H), 2.51 (d, *J* = 16.3 Hz, 1H), 1.59 (s, 3H). Spectroscopic data were in agreement with the literature [36].

### 3.4. 2-(1-Methyl-3-Oxoisoindolin-1-yl) Malonic Acid (***9***)

Isoindolinone **7** (400 mg, 1.44 mmol) was dissolved in CH_2_Cl_2_ (12 mL, 0.12M), and 2M NaOH in MeOH (6.5 mL) was added with stirring of the mixture at room temperature for 24 h. The solvent was removed under reduced pressure, and then the white solid was solubilized in water (2.5 mL) and washed with ethyl acetate. The aqueous layer was then acidified with 3N HCl and extracted with ethyl acetate 5 times, giving a white foam. Yield: 97% (350 mg). Used in the next reaction without further purification. IR (KBR): 3520, 1720, 1706, 1701 cm^−1^. HRMS (MALDI) *m/z:* [M + H]^+^ calcd for C_12_H_12_NO_5_ 250.07100, found: 250.07179. ^1^H NMR (CD_3_OD_,_ 250 MHz): δ 7.74–7.52 (m, 3H), 7.50 (t, *J* = 8.15 Hz, 1H), 4.02 (s, 1H) 1.70 (s, 3H). ^13^C-NMR (CD_3_OD_,_ 62.5 MHz): δ 170.7, 169.7, 168.7, 150.4, 132.3, 131.1, 129.1, 128.3, 122.6, 61.3, 29.8, 24.1.

### 3.5. 2-(1-Methyl-3-Oxoisoindolin-1-yl) Acetic Acid (***10***)

Compound **9** (300 mg, 1.20 mmol) was solubilized in CH_3_CN (5.2 mL, 0.23 M) and refluxed overnight. The solvent as removed under reduced pressure and rinsed with chloroform twice, affording a slightly yellow waxy oil. Yield: 99% (250 mg). IR (KBR): 3504, 1724, 1702 cm^−1^. HRMS (MALDI) *m/z:* [M + H]^+^ calcd for C_11_H_12_NO_3_ 206.08117, found: 206.08089. ^1^H NMR (CDCl_3,_ 300 MHz): δ 11.33 (br s, 1H, COOH), 8.79 (s, 1H, NH) 7.82 (d, *J* = 7.17 Hz, 1H), 7.59 (t, *J* = 7.23 Hz, 1H), 7.49–7.41 (m, 2H), 3.07 (d, *J* = 16.38 Hz, 1H), 2.53 (d, *J* = 16.38 Hz, 1H), 1.69 (s, 3H). ^13^C NMR (CDCl_3,_ 75 MHz): δ 174.5, 171.0, 151.8, 132.8, 130.3, 128.8, 124.4, 121.3, 60.3, 44.1, 24.7.

### 3.6. 3-Methyl-3-(2-(4-Methylpiperazin-1-yl) -2-Oxoethyl) Isoindolin-1-One (***12***)

Amide coupling was carried out according to a modified literature procedure [22]. To a solution of compound **10** (0.24 mmol, 1.0 eq.) in CH_2_Cl_2_ (2.8 mL), Et_3_N (0.48 mmol, 2.0 eq.), EDC.HCl (0.24 mmol, 1.0 eq.) and HOBt (0.24 mmol, 1.0 eq.), amine **11** (0.28 mmol, 1.2 eq.) was added, and the reaction mixture was allowed to stir at room temperature for 24 h. The mixture was diluted with dichloromethane and a small amount of K_2_CO_3_ was added. Purification by chromatography on silica gel (CHCl_3/_MeOH, 95:5) gave the product as an oil. Yield: 84% (58 mg). IR (neat): 3200, 1742, 1720, 1599 cm^−1^. HRMS (MALDI) *m/z:* [M + H]^+^ calcd. for C_16_H_22_N_3_O_2_ 288.17065, found: 288.17083. ^1^H NMR (CDCl_3,_ 300 MHz): δ 7.83 (d, *J* = 7.42 Hz, 1H), 7.55 (t, *J* = 7.54 Hz, 1H), 7.47–7.43 (m, 2H), 7.37 (d, *J* = 7.42 Hz, 1H), 3.78–3.76 (m, 1H), 3.75–3.74 (m, 1H), 3.55–3.38 (m, 2H), 2.98 (d, *J* = 16.38 Hz, 1H), 2.48–2.22 (m + s, 8H), 1.65 (s, 3H). ^13^C NMR (CDCl_3,_ 75 MHz): δ 168.8, 168.5, 152.4, 132.1, 131.2, 128.5, 124.4, 121.1, 59.6, 55.0, 54.8, 46.1, 45.5, 42.4, 41.6, 25.2.

### 3.7. 3-Methyl-3-(2-(4-Methylpiperazin-1-yl)Acetyl)-2-Phenylisoindolin-1-One (***13***)

Amide arylation was carried out according to a literature procedure [39]. A mixture of isoindolinone **12** (0.134 mmol), iodobenzene (0.268 mmol), CuI (0.0268 mmol) and K_2_CO_3_ (0.134 mmol) was dissolved in DMF (300 µL), evacuated and backfilled with nitrogen and then stirred under a nitrogen atmosphere at 140 °C for 36 h. The resulting pale-brown suspension was cooled to room temperature, diluted with NH_3_ and extracted twice with ethyl acetate. Purification by chromatography on silica gel (CHCl_3/_MeOH, 90:10) gave the product as brown solid. Yield: 81% (39 mg). Mp. 162 °C (dec.). IR (KBr): 1743, 1718, 1600 cm^−1^. HRMS (MALDI) *m/z* [M + H]^+^ calcd. for C_22_H_26_N_3_O_2_ 364.20195, found: 364.20107. ^1^H NMR (CDCl_3,_ 300 MHz): δ 7.92 (d, *J* = 7.31 Hz, 1H), 7.56–7.40 (m, 6H), 7.31–7.26 (m, 2H), 3.52 (t, *J* = 4.69 Hz, 2H), 3.24–3.13 (m, 1H), 3.09–3.01 (m, 1H), 2.75 (app q, *J* = 14.4 Hz, 2H), 2.30–2.28 (m, 3H), 2.23 (s, 3H), 2.10–2.07 (m, 1H), 1.73 (s, 3H). ^13^C NMR (CDCl_3,_ 75 MHz): δ 168.2, 167.2, 149.5, 135.8, 132.1, 131.4, 129.8, 129.7, 128.5, 124.3, 122.1, 65.7, 54.6, 54.5, 45.9, 45.7, 41.3, 40.5, 26.4.

### 3.8. 3-Methyl-3-(2-oxo-2-(1,4-Dioxa-8-Azaspiro [4.5] Decan-8-yl) Ethyl) Isoindolin-1-One (***15***)

Prepared following Yamada coupling [38]. To a solution of compound **10** (0.24 mmol, 1.0 eq) in CH_2_Cl_2_ (2.8 mL), Et_3_N (0.48 mmol, 2.0 eq.) and DEPC (0.24 mmol, 1.0 eq.), amine **14** (0.28 mmol, 1.2 eq.) was added, and the reaction mixture was allowed to stir at room temperature for 24 h. The reaction mixture was quenched with 1N HCl (2 mL) and extracted with dichloromethane. The organic layer was dried over MgSO_4_, evaporated in vacuo and then purified by chromatography on silica gel (Ethyl acetate/MeOH, 95:5) to give a white solid. Mp. 150–151 °C. Yield: 74% (59 mg). IR (KBr): 3203, 1747, 1720, 1600 cm^−1^. HRMS (MALDI) *m/z:* [M + Na]^+^ calcd. for C_18_H_22_N_2_O_3_Na 331.14718, found: 331.14957. ^1^H NMR (CDCl_3,_ 300 MHz): δ 7.83 (d, *J* = 7.41 Hz, 1H), 7.58–7.56 (m, 2H), 7.51–7.39 (m, 2H), 3.96 (s, 4H), 3.78–3.77 (m, 1H), 3.75–3.69 (m, 1H), 3.47–3.43 (m, 2H), 3.02 (d, *J* = 16.29 Hz, 1H), 2.36 (d, *J* = 16.29 Hz, 1H), 1.66–1.64 (m, 7H). ^13^C NMR (75 MHz, CDCl_3_): δ 168.8, 168.3, 152.3, 132.2, 131.1, 128.5, 124.4, 121.1, 106.7, 64.6, 59.7, 43.6, 42.2, 39.9, 35.6, 34.8, 25.0.

### 3.9. 2-(7-Chloro-1,8-Naphthyridin-2-yl)-3-Methyl-3-(2-oxo-2-(1,4-Dioxa-8-Azaspiro [4.5]Decan-8-yl) Ethyl) Isoindolin-1-One (3-Methyl-Pazinaclone, ***16***)

Amide arylation was carried out according to a literature procedure [40]. To a solution of isoindolinone **15** (60 mg, 0.18 mmol, 1.0 eq.) in 1,4-dioxane (1 mL), 9,9-dimethyl-4,5-bis(diphenylphosphino)xanthene (Xantphos) (12 mg, 10 mol%), 2,7-dichloro-1,8-naphthyridine (40 mg, 1.2 eq.), Pd (OAc)_2_ (5 mg, 10 mol%) and potassium carbonate (50 mg, 2.0 eq.) were evacuated and flushed with nitrogen. The mixture was stirred at 80 °C for 48 h. The mixture was allowed to cool and then partitioned between EtOAc and water. The organic extract was washed with brine, dried by MgSO_4_ and evaporated in *vacuo*. Purification by chromatography (CHCl_3/_MeOH, 98: 2) gave the product as white solid. Mp. 235–236 °C. Yield: 65% (58 mg). IR (neat): 1741, 1720, 1609, 1280 cm^−1^. HRMS (MALDI) *m/z:* [M + H]^+^ calcd. for C_26_H_26_ClN_4_O_4_ 493.16371, found: 493.16117. ^1^H NMR (CDCl_3,_ 250 MHz): δ 9.02 (d, *J* = 9.37 Hz, 1H), 8.17 (d, *J* = 8.90 Hz, 1H), 8.10 (d, *J* = 8.37 Hz, 1H), 7.95 (d, *J* = 7.61 Hz, 1H), 7.67 (t, *J* = 7.37 Hz, 1H), 7.57–7.46 (m, 2H), 7.41 (d, *J* = 8.05 Hz, 1H), 4.48 (d, *J* = 15.35 Hz, 1H), 3.87 (s, 4H), 3.67–3.44 (m, 4H), 3.33 (d, *J* = 15.35 Hz, 1H), 2.08 (s, 3H), 1.80–1.54 (m, 4H). ^13^C NMR (150 MHz, CDCl_3_): δ 169.9, 167.9, 155.7, 154.7, 154.4, 152.2, 139.6, 138.6, 134.7, 131.0, 128.9, 125.1, 122.6, 121.6, 119.4, 118.1, 107.5, 67.9, 64.9, 44.5, 40.2, 40.0, 36.1, 35.3, 30.4, 27.7.

### 3.10. 3-(2-Hydroxyethyl)-3-Methylisoindolin-1-One (***17***)

To a solution in anhydrous THF (4.80 mL) of compound **8** (210 mg, 0.96 mmol, 1.0 eq.), under nitrogen atmosphere in an ice bath, 2M LiBH_4_ (784 μL, 1.5 eq.) was added dropwise, and the reaction mixture was allowed to stir at room temperature overnight. The mixture was diluted with water (5 mL), and the aqueous phase was extracted three times with AcOEt (20 mL), giving a pale oil. Yield: 99% (180 mg). IR (neat): 3501, 1720, 1600 cm^−1^. HRMS (MALDI) *m*/*z:* [M + H]^+^ calcd. for C_11_H_14_NO_2_ 192.1000; found: 192.10191. ^1^H NMR (CDCl_3,_ 300 MHz): δ 7.80 (d, *J* = 7.76 Hz, 1H), 7.55 (t, *J* = 7.71 Hz, 1H), 7.46–7.26 (m, 3H), 3.75–3.73 (m, 2H), 2.84 (br s, 1H, OH), 2.11–1.99 (m, 2H), 1.56 (s, 3H). ^13^C NMR (CDCl_3,_ 100 MHz): δ 170.3, 125.7, 132.3, 131.0, 128.3, 124.0, 121.3, 61.5, 58.6, 41.7, 25.5.

### 3.11. 2-(1-Methyl-3-oxoisoindolin-1-yl)ethylmethanesulfonate (***18***)

In anhydrous DCM (240 μL) to a solution of alcohol **17** (90 mg, 0.48 mmol, 1.0 eq.) under nitrogen atmosphere, triethylamine (100 μL, 0.71 mmol, 1.5 eq.) and methansulfonyl chloride (43 μL, 0.55 mmol, 1.2 eq.) were added. The reaction mixture was stirred for 2 h at room temperature. *Method A workup*. The solvent was removed under reduced pressure, and the crude was diluted with AcOEt and washed with water recovering a pale oil. Yield: 95% (133 mg). *Method B work up*. The crude was directly purified by chromatography on Florisil (CHCl_3_/MeOH, 99:1). Yield: 73% (102 mg). IR (neat): 3471, 1720, 1400, 1201 cm^−1^. HRMS (ESI) *m/z:* [M + Na]^+^ calcd. for C_12_H_15_NO_4_SNa 292.0614; found: 292.0614. ^1^H NMR (CDCl_3,_ 400 MHz): δ 7.82 (d, *J* = 7.67 Hz, 1H), 7.61- 7.59 (m, 2H), 7.51–7.49 (m, 1H), 7,42–7.40 (m, 1H), 4.09–3.99 (m, 2H), 2.83 (s, 3H), 2.42–2.33 (m, 2H), 1.61 (s, 3H). ^13^C NMR (CDCl_3,_ 100 MHz): δ 170.2, 150.7, 132.7, 131.2, 128.8, 124.2, 121.5, 65.8, 60.4, 38.8, 37.3, 27.2.

### 3.12. 2-(1-Methyl-3-Oxoisoindolin-1-yl) Acetaldehyde (***19***)

Alcohol (70 mg, 0.36 mmol, 1.0 eq.) was dissolved in CH_2_Cl_2_ (1.6 mL), Dess–Martin periodinane (186 mg, 0.44 mmol, 1.2 eq.) was added and the mixture was stirred for 2 h at room temperature. Then, the suspension was diluted with dichloromethane (3 mL) and washed with 1N NaOH (1 mL), giving a pale oil, and it was purified by chromatography on silica gel (Ethyl acetate/MeOH, 95:5). Yield: 99% (68 mg). IR (neat): 3501, 1720, 1605 cm^−1^. HRMS (MALDI) *m/z:* [M + Na]^+^ calcd. for C_11_H_11_NO_2_Na 212.06820; found: 212.06764. ^1^H NMR (CDCl_3,_ 300 MHz): δ 9.64 (s, 1H, CHO), 7.84 (d, *J* = 7.62 Hz, 1H), 7.60 (t, *J* = 7.31 Hz, 1H), 7.51–7.40 (m, 3H), 3.13 (d, *J* = 17.31 Hz, 1H), 2.74 (d, *J* = 17.58 Hz 1H), 1.64 (s, 3H). ^13^C NMR (CDCl_3,_ 75 MHz): δ 199.8, 169.5, 151.1, 132.6, 130.8, 128.8, 124.4, 121.2, 59.1, 52.5, 25.9

### 3.13. 3-(2-(4-(3,4-Dimethylphenyl) Piperazin-1-yl) Ethyl)-3-Methylisoindolin-1-One (3-Methyl-PD172938, ***20***)

*Pathway A*. A mixture of compound **18** (120 mg, 0.44 mmol, 1.0 eq.) in CHCl_3_ (1.0 mL), triethylamine (60 μL, 0.44 mmol, 1.0 eq.) and 3,4 dimethylphenylpiperazine (100 mg, 0.53 mmol, 1.2 eq.) was stirred at 50 °C for 24 h. Purification by chromatography on silica gel (CHCl_3/_MeOH, 99/1) gave the product as a pink solid. Yield: 80% (127 mg).

*Pathway B*. Aldehyde **19** (60 mg, 0.30 mmol, 1.0 eq.) was dissolved in MeOH (2.4 mL), 3,4-(dimethyl) phenylpiperazine (62 mg, 0.30 mmol, 1.0 eq) was added and the mixture was stirred at room temperature for 5 h. Then, NaBH_4_ (32 mg, 1.5 eq.) was added and stirring was continued for 2 h. Purification by chromatography on silica gel (CHCl_3_/MeOH, 99:1) gave the product as a pink solid. Yield: 80% (87 mg). Mp. 57–58 °C. IR (neat): 3205, 1720 cm^−1^. HRMS (MALDI) *m/z:* [M + H]^+^ calcd. for C_23_H_30_N_3_O 364.23830, found: 364.23834. ^1^H NMR (CDCl_3,_ 300 MHz): δ 7.82 (d, *J* = 7.27 Hz, 1H, 7.56 (t, *J* = 7.48 Hz, 1H), 7.45 (t, *J* = 7.27 Hz, 1H), 7.37 (d, *J* = 7.27 Hz, 1H), 7.28 (s, 1H, NH), 7.00 (d, *J* = 7.75 Hz, 1H), 7.69 (m, 2H), 3.20 (m, 1H), 3.13 (s, 3H), 2.65 (m, 2H), 2.44 (m, 5H), 2.22 (s, 3H), 2.17 (s, 3H), 2.03 (m, 1H), 1.54 (s, 3H).^13^C NMR (CDCl_3,_ 100 MHz): δ 169.6, 152.5, 149.6, 137.2, 132.2, 131.3, 130.3, 128.4, 128.3, 124.2, 121.1, 118.4, 114.1, 61.2, 54.0, 53.5, 49.9, 36.4, 25.7, 20.3, 18.9.

### 3.14. Asymmetric Synthesis of (S)-PD172938 3-Methylated Analog, (S)-20

Dimethyl 2-(1-methyl-3-oxoisoindolin-1-yl)malonate *(S)-*7. Prepared by a literature-modified procedure. In a round bottom flask, 2-acetylbenzonitrile 6 (250 mg, 1.72 mmol, 1.0 eq.) was dissolved in CH_2_Cl_2_ (0.07M), then dimethylmalonate (750 μL, 5.16 mmol, 3 eq.), catalyst (*R,R*)-21 (107 mg, 10% mol) and K_3_PO_4_ (730 mg, 3.44 mmol, 2.0 eq.) were added and the mixture was stirred at room temperature for 24 h. After filtration of the inorganic salt, the solvent was removed, and the crude was purified by chromatography on silica gel (Hexane/Ethyl acetate, 50:50) affording a white solid. Yield: 84% (400 mg). Ee: 44%, Chiracel OD-H Hex/*i*-PrOH 80/20, 0.6 mL/min, λ 254 nm, t_minor_: 15.3 min and t_major_: 16.7 min. Crystallization procedure: the sample was dissolved in a mixture of CH_2_Cl_2_/Hexane (12 mL, 1/4) at room temperature and then left at −20 °C for 72 h. The enantio-enriched product was recovered as waxy oil. Ee: 94%. [α]_D_ 21 °C: −101.6 (c 0.3, CHCl_3_). Yield: 47% (188 mg).

2-(1-Methyl-3-oxoisoindolin-1-yl) malonic acid *(S)-*8 and 2-(1-Methyl-3-oxoisoindolin-1-yl)acetic acid *(S)-*9. Prepared as reported for racemic compounds, starting from 180 mg (0.65 mmol) of enantioenriched compound *(S)-*7. Used without further purification. Yield after two steps: 99% (135 mg). The enantiomeric excess of the acid was determined after derivatization into methylester derivative *(S)-*8.

Methyl 2-(1-methyl-3-oxoisoindolin-1-yl) acetate, *(S)-*8. Isoindolinone *(S)-*10 (135 mg, 0.64 mmol, 1.0 eq.) was dissolved in acetone (8.5 mL), then potassium carbonate (51 mg, 0.37 mmol, 0.6 eq.) and dimethylsulphate (110 μL, 1.10 mmol, 1.5 eq.) were added, keeping the reaction at 50 °C under stirring for 2 h. After filtration of inorganic salt, the crude was purified by chromatography on silica gel (Hexane/Ethyl acetate 50:50) affording the ester as a pale oil. Yield: 98% (140 mg). Ee: 94%. Chiracel IE-3 Hex/*i*-PrOH 70/30, 0.6 mL/min, λ 254 nm, t_minor_: 28.5 min and t_major_: 35.9 min. [α]_D_19 °C: −66.1 (c 0.3, CHCl_3_). Spectroscopic data are in agreement with racemate [36].

3-(2-Hydroxyethyl)-3-methylisoindolin-1-one *(S)-*17 and 2-(1-Methyl-3-oxoisoindolin-1-yl)ethylmethanesulfonate *(S)-*18 were prepared as described for racemic compounds, starting from 140 mg (0.64 mmol) of enantioenriched compound *(S)-*8 and used without further purification.

2-(1-Methyl-3-oxoisoindolin-1-yl)acetaldehyde *(S)-*19 were prepared as described for racemic compound, starting from 35 mg (0.18 mmol) of enantoenriched compound *(S)-*17. Purification by chromatography on silica gel (Ethyl acetate/MeOH 95:5) gave a pale oil. Yield: 99% (34 mg). [α]_D_ 25 °C: −52.4 (c 0.8, CHCl_3_).

3-(2-(4-(3,4-Dimethylphenyl)piperazin-1-yl)ethyl)-3-methylisoindolin-1-one *(S)-*20. *Pathway A. (S)-*18 (60 mg, 0.22 mmol, 1.0 eq.) was dissolved in CHCl_3_ (350 μL), then triethylamine (30 μL, 0.22 mmol, 1.0 eq.) and 3,4-(dimethyl) phenylpiperazine (50 mg, 0.53 mmol, 1.2 eq.) were added and the mixture was stirred at 50 °C for 24 h. Purification by chromatography on silica gel (CHCl_3_/MeOH 99:1) gave the product as pink wax oil. Yield: 80% (63 mg). Ee: 94%. *Pathway B.* Aldehyde *(S)-***19** (30 mg, 0.15 mmol, 1.0 eq.) was dissolved in MeOH (1.2 mL), 3,4-(dimethyl) phenylpiperazine (31 mg, 0.15 mmol, 1 eq.) was added and the mixture was stirred at room temperature for 5 h. Then, NaBH_4_ (16 mg, 1.5 eq.) was added and stirring was continued for 2 h. Purification by chromatography on silica gel (CHCl_3_/MeOH 99:1) gave the product as pink wax oil. Yield: 80% (43 mg). Ee: 94%. Chiracel OD-H Hex/*i*-PrOH 80/20, 0.6 mL/min, λ 254 nm, t_minor_: 14.9 min and t_major_: 24.3 min. HRMS (MALDI) *m/z:* [M + H]^+^ calcd. for C_23_H_30_N_3_O 364.23835; found: 364.23834. [α]_D_ 19°C: −25.0 (c 0.3, CHCl_3_). Spectroscopic data were found in agreement with racemate, as reported above.

## 4. Conclusions

Herein we report the first racemic and asymmetric synthesis of novel 3-methylated analogs of valuable biologically active isoindolinones, bearing a tetrasubstituted stereocenter. In particular, 3-methyl-Pazinaclone was obtained in five steps and 39% overall yield. 3-Methyl-PD172938 was obtained in seven steps and about 60% overall yield. Since the (*S*) enantiomer of PD172938 has been reported to show superior biological activity [21], asymmetric synthesis of its 3-methylated analog was also developed based on asymmetric phase transfer catalysis cascade process. The target product was obtained with high enantiopurity (94% ee) in eight steps and about 30% overall yield.
